# Breaking the silence of sharing data in medical research

**DOI:** 10.1371/journal.pone.0301917

**Published:** 2024-05-29

**Authors:** Henian Chen, Yayi Zhao, Biwei Cao, Donna J. Petersen, Matthew J. Valente, Weiliang Cen

**Affiliations:** College of Public Health, University of South Florida, Tampa, FL, United States of America; National Center for Global Health and Medicine, JAPAN

## Abstract

Data sharing is highly advocated in the scientific community, with numerous organizations, funding agencies, and journals promoting transparency and collaboration. However, limited research exists on actual data sharing practices. We conducted a comprehensive analysis of the intent to share individual participant data (IPD) in a total of 313,990 studies encompassing clinical trials and observational studies obtained from ClinicalTrials.gov, spanning the period from 2000 to 2023. Our study found that only 10.3% of principal investigators (PIs) expressed intent to share IPD. Clinical trials were more likely to share data than observational studies (odds ratio, OR = 1.98, 95% CI: 1.92–2.04). Large sample size studies were 1.69 times more likely to share data than small ones (95% CI: 1.65–1.73). Studies registered after 2018 were 1.6 times more likely to share data (95% CI: 1.57–1.64) than before 2019. NIH and other US Federal agency-funded studies had 1.49 times higher odds of sharing data (95% CI: 1.43–1.55) than other funders. USA-based studies were 1.53 times more likely to share data (95% CI: 1.49–1.57) than out of USA. Biological trials were 1.58 times more likely to share data than drug and other trials (95% CI: 1.51–1.66). Phase III trials had the highest odds, 2.47 times, of sharing data (95% CI: 2.38–2.56) than non-Phase III trials.

## Introduction

Individual Participant Data (IPD) refers to the detailed data collected on individual participants in a research study, such as their demographic information, medical history, treatment interventions, and outcomes. Unlike aggregated data, which summarizes the results at a group-level, IPD allows for more in-depth analyses and individual-level investigations [1].The term IPD sharing refers to the practice of making study data at the individual-level available to researchers who were not part of the original study team. In January 2016, the International Committee of Medical Journal Editors (ICMJE) published a proposal stating that, as a condition of consideration for publication, authors of clinical trials should be required to share deidentified IPD underlying the results presented in their manuscripts within six months of publication. On July 1, 2018, the ICMJE implemented a new policy requiring manuscripts reporting the results of clinical trials to include a data sharing statement. This policy aimed to encourage authors to explicitly address data sharing in their publications [2, 3]. In December 2015, ClinicalTrials.gov added 2 optional registration fields for disclosing trialists’ intentions about IPD sharing: 1) plan to share IPD? and 2) available IPD/information type [4]. ClinicalTrials.gov added additional subfields with greater structure in late June 2017 to facilitate more complete disclosure of IPD-sharing plans as required by the ICMJE [4]. From January 1, 2019, the ICMJE extended its data sharing policy to include a requirement for clinical trials enrolling participants. The policy mandated that trials registering participants must include a data sharing plan in their trial registration [3]. Further, on January 25th, 2023, the National Institutes of Health (NIH) issued a new Data Management and Sharing Policy (DMS) which applies to all research funded or conducted by the NIH that results in the generation of scientific data. The DMS policy requires a DMS plan outlining how scientific data will be shared and compliance achieved [5]. These milestones reflect a growing recognition of and emphasis on the importance of sharing IPD in scientific research. However, despite the increased emphasis on the importance of sharing IPD in scientific research and clear directives to do so, the practice of data sharing from registered clinical trials has not increased commensurate with this goal.

Sharing IPD has several advantages including [1, 3, 4, 6–8]: 1) Sharing IPD allows researchers and the wider scientific community to access, analyze and scrutinize the original data behind research findings. 2) Shared IPD can be used to explore additional research questions beyond those initially investigated in the original studies. 3) IPD sharing facilitates meta-analyses and systematic reviews by providing access to individual-level data across multiple studies. 4) Sharing IPD encourages collaboration among researchers, institutions, and data custodians. 5) IPD pooling from multiple studies can lead to larger sample sizes and increased statistical power, allowing for more robust analyses, and better precision in estimating treatment effects. 6) Shared IPD can contribute to a broader evidence base, allowing healthcare providers and policymakers to make more informed decisions, ultimately leading to improved patient care and better public health outcomes. 7) Sharing IPD aligns with ethical principles of maximizing the value of data collected from research participants. Mello et al [9] reported that few clinical trial participants had strong concerns about the risks associated with data sharing. In fact, most participants expressed their willingness to have their data shared for a variety of uses.

The scientific community is supportive of the practice of data sharing. Many organizations, funding agencies, and journals have recognized the importance of data sharing and have implemented policies and initiatives to promote transparency and collaboration. Numerous papers have addressed and advocated for the sharing of IPD for scientific research [1, 4, 6–18] though data on actual data sharing is limited. There is a lack of comprehensive data regarding the current state of IPD sharing, highlighting the need for further investigation in this area. We conducted a comprehensive analysis of 313,990 studies, including both clinical trials and observational studies, obtained from ClinicalTrials.gov. The study spanned a period from 2000 to 2023.

## Methods

ClinicalTrials.gov managed by the US NIH National Library of Medicine (NLM), is currently the largest clinical trial registry globally. Since its launch in 2000, it now encompasses more than 450,000 registered studies, including both interventional and observational studies, conducted across 220 countries [2]. To gather data, we conducted a comprehensive search on ClinicalTrials.gov [2], encompassing all studies registered prior to June 13, 2023, utilizing the Clinical Trials Transformation Initiative (CTTI) Aggregate Analysis of Clinical Trials (AACT). The CTTI AACT website (https://aact.ctti-clinicaltrials.org/snapshots) archives a static copy of the ClinicalTrials.gov database on the first day of each month. We retrieved and downloaded a total of 450,564 studies. The intention regarding IPD sharing is determined by the responses to the question: "Do you plan to share individual participant data (IPD)?" The possible answers to this question include "yes”, “no”, “undecided," or "no response" (answer not provided). ClinicalTrials.gov introduced this IPD question in December 2015 [4], therefore we utilized the IPD variable information for all studies registered or completed after December 2015. All studies that were registered or completed before December 2015 had the opportunity to retrospectively answer this IPD question. Therefore, for studies registered or completed before December 2015 or missing date of registration we only included those of which provided answers of “yes”, “no”, or “undecided” to the IPD sharing question retrospectively and excluded the studies that did not provide a response (answer not provided).

We use the following equation to calculate the rate of agreed to share data rate (R) for studies both registered and completed before December 2015 or missing date of registration: R = n1(yes) / N(total) where N(total) = n1(yes) + n2(no) + n3(undecided). The following equation was used to calculate the R for studies 1) registered after December 2015, and 2) registered before December 2015 and completed after December 2015: R = n1(yes) / N(total) where N(total) = n1(yes) + n2(no) + n3(undecided) + n4(no response).

For each study, we collected various information including details about funders, years, locations, enrollment (sample size), classification as a clinical trial or an observational study. We categorized them as follows: "NIH/US Fed": If the study listed at least one NIH institute or center, or any other US Federal agency, as the sponsor. "Industry": If the study was not classified as NIH/US Fed, but listed at least one company as the lead sponsor or collaborator. "All others": If the study was funded by individuals, universities, community-based organizations, or foundations. For clinical trials specifically, we obtained additional information such as the phase of the trial (ranging from phase I to phase IV), the primary purpose of the treatment, and details about the interventions used. The classification of clinical trial phases from phase I to phase IV was based on Food and Drug Administration (FDA) guidelines and definitions provided in the ClinicalTrials.gov glossary of common site terms.

To analyze the data, we calculated the overall rates and sample sizes for the four response options (yes, no, undecided, or no response) to the IPD sharing question. We also examined these rates within various subgroups, including clinical trials vs. observational studies, funders, years, interventions, and phases. To determine significant differences in rates, we employed chi-square tests. Additionally, we conducted trend tests to assess rate changes over time. Furthermore, logistic regression models were utilized to examine associations between the agreement to share IPD and relevant factors. These models allowed us to assess the relationships between "agreed to share IPD" and various factors of interest. Odds ratios were estimated from logistic regression models.

## Results

### 313,990 studies registered from ClinicalTrials.gov

**Fig 1:**
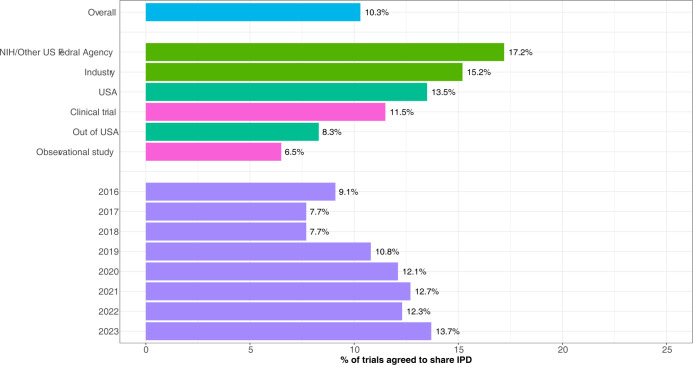
Percent of all studies agreed to share IPD by study characteristics. Overall, these findings provide a comprehensive overview of the extent of intent to share IPD across a vast number of studies, highlighting variations based on study characteristics and other influencing factors.

We conducted a comprehensive analysis of the intent to share IPD in a total of 313,990 studies encompassing clinical trials and observational studies obtained from ClinicalTrials.gov, with the majority of the studies spanning the period from 2000 to 2023. There were 12,626 studies that initially registered and completed prior to December 2015, when specifying a data sharing plan was not possible, that retrospectively answered ‘Yes’, ‘No’ or ‘Undecided’ to the question about an IPD data sharing plan. A total of 3,622 (28.7%) studies answered ‘Yes’, 7,451 (59.0%) answered ‘No’ and 1,553 (12.3%) answered ‘Undecided’. A total of 32,210 PIs (10.3%) indicated their intention to share IPD. Of the analyzed studies, 237,147 (75.5%) were classified as clinical trials, while 76,835 (24.5%) were categorized as observational studies. Among the clinical trials, a total of 27,232 PIs (11.5%) indicated their intention to share IPD. In the case of observational studies, 4,978 PIs (6.5%) expressed a positive inclination to share IPD. Regarding sponsorship, the rates of PIs indicating an intent to share IPD were 17% for studies sponsored by the NIH/other US Federal agencies, and 15% for studies sponsored by industry, and 8% for studies sponsored by Other. Regarding location, 14% of PIs in the United States (US) indicated an intent to share data compared to 8.3% for studies conducted outside the US. Additionally, we investigated the rates of "yes" responses based on the registered years of the studies. The rates varied, with a range of 7.7% to 14% for studies registered between 2016 and 2023. In the context of clinical trials, we observed differing rates of "yes" responses based on the trial characteristics (see Table 1 and Fig 1).

**Table 1:** Trial characteristics and whether study agreed to sharing IPD.

Characteristic		Sharing IPD			
	Sub-group sample size	Yes	No	Undecided	No response
**Among all studies**					
**Overall, N = 313,990**		32,210 (10.3%)	133,761 (42.6%)	35,862 (11.4%)	112,157 (35.7%)
**Study type**					
Clinical trials	237,147	27,232 (11.5%)	104,522 (44.1%)	22,172 (9.3%)	83,221 (35.1%)
Observational	76,835	4,978 (6.5%)	29,239 (38.1%)	13,690 (17.8%)	28,928 (37.6%)
Others	8	0 (0%)	0 (0%)	0 (0%)	8 (100%)
**Funder**					
NIH/Other USA Federal Agency	25,002	4,300 (17.2%)	9,198 (36.8%)	1,545 (6.2%)	9,959 (39.8%)
Industry	79,263	12,043 (15.2%)	31,770 (40.1%)	6,562 (8.3%)	28,888 (36.4%)
Others	209,725	15,867 (7.6%)	92,793 (44.2%)	27,755 (13.2%)	73,310 (35.0%)
**Location**					
USA	104,325	14,133 (13.5%)	45,460 (43.6%)	7,222 (6.9%)	37,510 (36.0%)
Out of USA	177,642	14,820 (8.3%)	74,589 (42.0%)	24,099 (13.6%)	64,134 (36.1%)
Unknown	32,023	3,257 (10.2%)	13,712 (42.8%)	4,541 (14.2%)	10,513 (32.8%)
**Year**					
Started and completed on or before 2015	12,626	3,622 (28.7%)	7,451 (59.0%)	1,553 (12.3%)	—-
Started on or before 2015 and completed post 2015	66,920	3,317 (5.0%)	13,467 (20.1%)	4,590 (6.9%)	45,546 (68.1%)
Started on 2016	26,191	2,380 (9.1%)	11,411 (43.6%)	3,898 (14.9%)	8,502 (32.5%)
Started on 2017	27,531	2,110 (7.7%)	13,690 (49.7%)	4,361 (15.8%)	7,370 (26.8%)
Started on 2018	29,495	2,281 (7.7%)	13,435 (45.6%)	4,695 (15.9%)	9,084 (30.8%)
Started on 2019	31,029	3,365 (10.8%)	15,290 (49.3%)	3,829 (12.3%)	8,545 (27.5%)
Started on 2020	32,111	3,878 (12.1%)	15,547 (48.4%)	3,748 (11.7%)	8,938 (27.8%)
Started on 2021	35,572	4,500 (12.7%)	17,567 (49.4%)	3,685 (10.4%)	9,820 (27.6%)
Started on 2022	32,655	4,032 (12.3%)	15,999 (49.0%)	3,509 (10.7%)	9,115 (27.9%)
Started on 2023	19,280	2,641 (13.7%)	9,629 (49.9%)	1,894 (9.8%)	5,116 (26.5%)
To be started post 2023	560	82 (14.6%)	264 (47.1%)	93 (16.6%)	121 (21.6%)
Unknown	20	2 (10%)	11 (55%)	7 (35%)	0 (0%)
**Among clinical trials**					
**Overall, N = 237,147**		27,232 (11.5%)	104,522 (44.1%)	22,172 (9.4%)	83,221 (35.1%)
**Primary purpose**					
Treatment	147,122	17,559 (11.9%)	61,513 (41.8%)	13,666 (9.3%)	54,384 (37.0%)
Non-Treatment	88,907	9,615 (10.8%)	42,717 (48.0%)	8,402 (9.5%)	28,173 (31.7%)
Unknown	1,118	58 (5.2%)	292 (26.1%)	104 (9.3%)	664 (59.4%)
**Phase**					
Phase 1	35,490	4,229 (11.9%)	14,094 (39.7%)	2,746 (7.7%)	14,421 (40.6%)
Phase 2	35,829	4,927 (13.8%)	13,351 (37.3%)	2,978 (8.3%)	14,573 (40.7%)
Phase 3	19,022	4,393 (23.1%)	5,741 (30.2%)	1,523 (8.0%)	7,365 (38.7%)
Phase 4	16,727	2,000 (12.0%)	7,091 (42.4%)	1,683 (10.1%)	5,953 (35.6%)
Unknown	130,079	11,683 (9.0%)	64,245 (49.4%)	13,242 (10.2%)	40,909 (31.4%)
**Intervention type**					
Biological	13,449	2,409 (17.9%)	4,600 (34.2%)	1,067 (7.9%)	5,373 (40.0%)
Drug	88,916	12,372 (13.9%)	34,525 (38.8%)	7,687 (8.6%)	34,332 (38.6%)
Others	134,782	12,451 (9.2%)	65,397 (48.5%)	13,418 (10.0%)	43,516 (32.3%)

*Chi-squared tests were used to compare the percentages of studies agreed to share IPD between studies funded by NIH/other USA federal agency vs. studies funded by industry (p-value<0.0001), studies performed in USA vs. studies performed outside of USA (p-value<0.0001), clinical trials with primary purpose being treatment vs. non-treatment (p-value<0.0001), clinical trials with phases of 1, 2, 3, and 4 (p-value<0.0001), and clinical trials using biological vs. drug as type of intervention (p-value<0.0001). **Cochran-Armitage tests were used assess the trend in the percentages of studies agreed to share IPD across the years for studies registered from 2016 to 2023 (p-trend<0.0001).

### Factors related to sharing IPD

Table 2 illustrates the relationship between these factors and the willingness to share IPD. The following factors were found to be significantly associated with the likelihood of sharing data: study types, sample sizes, funders, registration years, locations, interventions, and trial phases. Clinical trials had significantly higher odds of sharing data compared to observational studies, with an odds ratio (OR) of 1.98 (95% confidence interval [CI]: 1.92–2.04). This indicates that the odds of sharing data in clinical trials were about twice as high as in observational studies. Studies with large sample sizes had 1.69 times higher odds of sharing data compared to those with small sample sizes (OR = 1.69, 95% CI: 1.65–1.73). Similarly, studies registered after 2018 had 1.6 times higher odds of sharing data compared to studies registered before 2019 (OR = 1.60, 95% CI: 1.57–1.64). Furthermore, studies funded by the NIH and other US Federal agencies had 1.49 times higher odds of sharing data compared to studies funded by other sources (OR = 1.49, 95% CI: 1.43–1.55). Studies conducted in the US had 1.53 times higher odds of sharing data compared to studies conducted outside of the US (OR = 1.53, 95% CI: 1.49–1.57). Additionally, biological trials had 1.58 times higher odds of sharing data compared to drug and other trials (OR = 1.58, 95% CI: 1.51–1.66). Phase III trials had 2.47 times higher odds of sharing data compared to trials in other phases (OR = 2.47, 95% CI: 2.38–2.56).

**Table 2:** Odds ratio and 95% confidence interval for agreeing to IPD sharing by study characteristics.

Characteristic	Odds ratio (95% confidence interval)	p-value
Clinical trial	1.98 (1.92–2.04)	<0.0001
NIH-funded	1.49 (1.43–1.55)	<0.0001
USA	1.53 (1.49–1.57)	<0.0001
Large sample size	1.69 (1.65–1.73)	<0.0001
After 2018	1.60 (1.57–1.64)	<0.0001
Phase III trial	2.47 (2.38–2.56)	<0.0001
Biological trial	1.58 (1.51–1.66)	<0.0001

Note: Odds ratios were derived from logistic regression models with the reference groups; Clinical trial vs., observational study; NIH-funded vs., other funder; USA vs., out of USA; large sample size means planed sample size ≥ median planed sample size comparing with small sample size (planed sample size ≤ median planed sample size) group; we compared registered years 2000–2018 and 2019–2023 because from January 1, 2019 the International Committee of Medical Journal Editors (ICMJE) extended its data sharing policy on January 1, 2019, and this extension required clinical trials enrolling participants to include a data sharing plan in their trial registration; Phase III trial vs., non-Phase III trial; and Biological trial vs., non-Biological trial.

## Discussion

The intention of authors to share data does not always align with their actual data sharing practices, as noted by Watson [19, 20]. Gabelica et al. [21] conducted a comprehensive study involving 1,792 peer-reviewed publications in which authors expressed their willingness to share data upon reasonable request. Surprisingly, 93.1% (n = 1669) of the corresponding authors either declined or did not respond to requests for raw data, and only 6.9% (n = 123) provided the data in a usable format. It seems that data-availability statements are of little value because many of the data sets are never actually made accessible. It is important to acknowledge that the rate of agreement to share data should ideally be higher for publications compared to registrations as authors are required to make a commitment to data sharing to publish research papers.

The U.S. government has made efforts to encourage companies, universities, and other institutions conducting clinical trials to record and report their results in a federal database. However, compliance with this requirement has been inconsistent, even after a 2007 law made it mandatory for registered trials. Many sponsors still choose to ignore the reporting requirement, while federal officials have done little to enforce the law [22, 23]. Given this lack of compliance with reporting trial results, it becomes increasingly challenging to expect these same organizations to willingly share their IPD. In 2022, Sim et al [24]. proposed that the NIH should take a leading role in promoting scientific data sharing, particularly focusing on clinical trial data sharing. NIH’s recent DSM policy (effective January 25th, 2023) marks a big step toward open data practices as NIH is the largest funder of biomedical research in the world.

Given the myriad justifications in support of data sharing, it is time for the scientific community to hold itself accountable and take deliberate steps to rectify this unacceptable situation. Statements of intentions are not enough; PIs, as a condition of registering clinical trials and observational studies should be required to commit to sharing IPD and to describe specifically how and when they intend to do so. Editors should hold authors accountable by requiring them to include the same information in all accepted manuscripts, providing direct access for other scientists to the de-identified IPD reported in the publication. As scientists in the field of health, we have a responsibility to create efficiencies that can accelerate the discovery and sharing of knowledge for the benefit of patients and communities. As a society, we have neither the time nor the money to squander the precious resource of already gathered data in pursuit of improvements in the quality of life for everyone.

While sharing data undoubtedly promotes transparency and collaboration in scientific research, it also raises several important considerations. One such concern is the protection of participant privacy and confidentiality, particularly in sensitive research areas. Additionally, there may be challenges in ensuring the accuracy and integrity of shared data, as well as concerns regarding appropriate attribution and recognition for the original researchers. Addressing these potential concerns is crucial for establishing responsible data sharing practices and fostering trust within the scientific community.

This study has several limitations. First, we lack information regarding the reasons why IPD were not shared, as such data is not available. Second, not all registered studies are completed or carried out to fruition, rendering the initial intention to share IPD less significant. Third, ClinicalTrials.gov introduced the IPD question in December 2015. Studies registered or completed before this date had the opportunity to retrospectively answer the IPD question. Consequently, our analyses only included studies that provided retrospective responses of ’yes’, ’no’, or ’undecided’ to the IPD sharing question, while excluding those that did not respond (answer not provided). This may result in an overestimation of the rate of expressed intent to share IPD.

It is important to note that the IPD question within ClinicalTrials.gov is not a mandatory item. As a result, many trialists choose to provide no response (answer not provided). We recommend that this question be made mandatory. Additionally, we suggest that the IPD question should be modified to offer only two response options (yes or no), removing the "undecided" choice, and requiring a justification for a refusal to share data. These modifications would facilitate clearer reporting and assessment of the intent to share IPD.

## Supporting information

S1 Data (CSV)

S2 Data (CSV)

S3 Data (CSV)

S4 Data (CSV)

S1 File (TXT)
